# Multiple risk factors for persistent HBV viraemia in an adult receiving nucleos/tide analogue therapy

**DOI:** 10.1136/sextrans-2024-056168

**Published:** 2024-07-26

**Authors:** Sheila F Lumley, Maeve Barlow, Khadija Said Mohammed, Emily Martyn, Elizabeth Waddilove, Marion Delphin, Daisy Jennings, Haiting Chai, Agnes Kemper, Joy Ko, Azim Ansari, Douglas Macdonald, Samreen Ijaz, Indrajit Ghosh, Stuart Flanagan, Philippa C Matthews

**Affiliations:** 1 Nuffield Department of Medicine, University of Oxford, Oxford, UK; 2 Department of Infectious Diseases and Microbiology, Oxford University Hospitals NHS Foundation Trust; 3 Central North West London NHS Foundation Trust, London UK; 4 The Francis Crick Institute, London, UK; 5 University College London, London, UK; 6 Royal Free Hospital, London, UK; 7 Blood Borne Virus Unit, UK Health Security Agency, Colindale, London

**Keywords:** hepatitis b virus, HBV, resistance, breakthrough, therapy, case report, nucleos/tide analogue

## Abstract

Diagnosing and treating chronic hepatitis B virus (HBV) infection are key interventions to support progress towards elimination of viral hepatitis by 2030. Although nucleos/tide analogue (NA) therapy is typically highly effective, challenges remain for viral load (VL) suppression, including medication access, incomplete adherence, and drug resistance. We present a case of a long-term HBV and HIV co-infected adult prescribed sequential NA therapy regimens, with episodes of breakthrough viraemia. Multiple factors contribute to virologic breakthrough, including exposure to old NA agents, initial high HBV VL, therapy interruptions, intercurrent illnesses and potential contribution from resistance mutations. The case underscores the importance of individualized treatment approaches and adherence support in achieving HBV suppression. Furthermore, it emphasizes the need for improved clinical pathways addressing education, support, and access to care, particularly for marginalized populations. Comprehensive data collection inclusive of underrepresented individuals is crucial for maintaining retention in the care cascade, and informing effective interventions.

## Background

In line with international goals to eliminate viral hepatitis as a public health threat by 2030 [1], there is a global drive to diagnose,treat and prevent hepatitis B virus (HBV) infection. Nucleos/tide analogue (NA) agents suppress HBV DNA to below quantifiable thresholds in the majority of people receiving treatment. However, viraemia persists in a proportion of those offered treatment (up to 20% after one year in a recent population analysis [2]), attributed to a range of influences which may include drug resistance due to the selection of polymorphisms (RAMs) in the viral reverse transcriptase (RT). Resistance is predictably selected by exposure to lamivudine (3TC), which also influences susceptibility to entecavir (ETV) and adefovir (ADV) [3]. In contrast, resistance to tenofovir (TFV) is uncommon due to a high genetic barrier [4,5].

We here describe the case of an adult in whom HBV viraemia has not been consistently suppressed on treatment, to highlight a vulnerable population with risk factors for virologic breakthrough.

### HBV case report: Presentation, Investigations and Treatment

**Figure 1:**
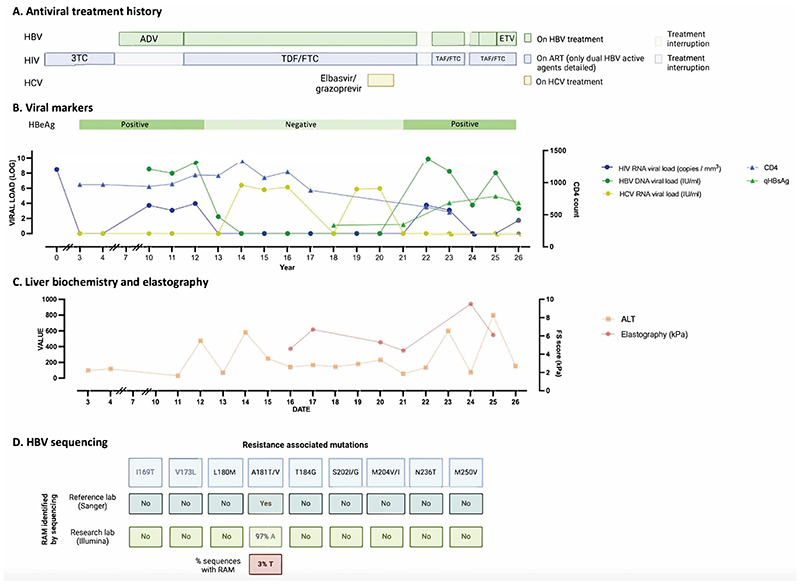
Summary details of non-suppression of HBV VL in an adult prescribed antiviral therapy. A: Timeline showing antiviral treatment for HBV, HIV and HCV, showing years since diagnosis on x axis. B: Timeline showing trends in viral loads for HBV, HIV and HCV, in addition to CD4+ count, quantitative HBsAg and HBeAg status (NB x-axis not to scale). C: Timeline showing ALT and liver elastography scores (NB x-axis not to scale). D: Resistance associated mutations (RAMs) listed in EASL guidance[7] mapped to the HBV polymorphisms identified in HBV sequenced from this individual through Sanger and Illumina sequencing.

An adult male received treatment for HBV and HIV co-infection over a period of 26 years in a central London clinic (Figure 1). We retrospectively reviewed data from his routine clinical records. Written informed consent was obtained; additional samples and data were collected with ethical approval (ref. 11/LO/0421).

At initial diagnosis HBV VL was 8.5 log_10_ IU/ml and HBeAg was positive. He screened negative for HDV infection. Antiretroviral therapy (ART) was commenced, with HIV suppression from 3.7 log_10_ RNA copies/ml to undetectable. Subsequently HBV was diagnosed and HBV VL progressively suppressed on HBV-active regimens containing 3TC (from baseline), switched to ADV (year 8), and then TFV (year 12), prescribed in line with changing guidelines. HBeAg was undetectable by year 13 (although he did not develop anti-HBe). He received successful intercurrent treatment for HCV infection..

Due to illness and emergency hospital admissions unrelated to blood borne virus infection, he was unable to sustain NA treatment, with documented gaps at intervals between years 22 and 25. HIV and HBV rebounded, with associated liver inflammation (peak ALT 902 IU/L), and HBeAg status reverted to positive. On reinstating HBV-active ART (including TFV and emtricitabine), HIV VL suppressed within 1 month, but HBV VL remained persistently elevated between 3.3-9.9 log_10_ IU/ml (Figure 1).

A clinical diagnostic laboratory identified the presence of a resistance mutation at position 181 in the HBV RT sequence (A181T) and reported potential drug resistance to 3TC, ADV and telbivudine. HBV Illumina sequence analysis demonstrated dual infection with HBV genotypes A and G (supplementary methods), and confirmed the A181T polymorphism, although only in a minority of quasispecies (Figure 1B). Alongside support to optimise adherence, ETV was added (in keeping with clinical guidelines [6][7]), followed by an HBV virologic response to 2.4 log_10_ IU/ml after 1 month and 3.2 log_10_ IU/ml at 4 months.

## Discussion

A number of factors can collectively reduce the success of NA treatment for HBV, including high baseline VL, HIV coinfection, exposure to historic regimens with low genetic barrier to resistance, therapy interruptions, physical and mental health comorbidities and complex barriers to continuity of care. These are interrelated and are likely to have a cumulative influence.

Individuals with these characteristics may be in vulnerable or marginalised groups, are unlikely to be eligible for clinical studies, and may experience social stigma and discrimination. These influences mitigate against their inclusion in laboratory data, clinical cohorts and trials [4]. There is a need for focus on equitable representation of the real-world challenges of life-long therapy to fill this current ‘blind spot’ (*further discussed in our Editorial*).

The contribution of drug resistance is doubtful in our patient, as A181T alone is not a recognised cause of TDF resistance [8]; a resistant phenotype would typically require multiple associated RAMs. More clinical and *in vitro* data are still needed to ascertain the relative contributions of different RAMs, alone and in combination. Adding HBV active agents, and providing adherence support, resulted in progressive reduction in viraemia over time to <2000 IU/ml, but not to undetectable.

Persistent HBV viraemia poses risks for transmission, and long-term inflammatory/fibrotic liver disease (indicated by elevated ALT in this case), highlighting the need for intervention. Service improvements should focus on flexible, patient-centric access to information, consistent supplies of medication, avoiding out-of-pocket costs, and providing access to peer support, particularly for those coping with other health and/or social challenges and for whom there are barriers to care access. As HBV treatment eligibility expands and we work towards elimination targets, research is needed to better determine the factors that contribute to the presence and impact of persistent viraemia, and to optimise surveillance and clinical intervention.

## Supplementary Material

Supplementary Table 1

## Key messages

- Current knowledge - HBV viraemia may persist or rebound in adults receiving NA treatment; a number of factors may collectively reduce the success of treatment in mediating long-term HBV suppression.
- What this study adds - we illustrate a complex case where multiple factors contribute to non-suppressed HBV VL on NA treatment.
- Implications - we raise awareness of diverse factors which may be contributing to non-suppressed HBV VL, and advocate for improved clinical pathways and comprehensive data collection to improve care and address knowledge gaps surrounding VL non-suppression on treatment.
