# Supplementary material for: Multiple risk factors for persistent HBV viraemia in an adult receiving nucleos/tide analogue therapy
Source: Sex Transm Infect. Author manuscript; Available in PMC 2024 Dec 16. (PMC7617237; doi:10.1136/sextrans-2024-056168)
Supplement: Supplementary Table 1 [file EMS201731-supplement-Supplementary_Table_1.pdf]

SUPPLEMENTARY MATERIAL

Supplementary methods

Sanger sequencing and resistance reporting was undertaken by University College London Hospitals clinical diagnostic virology laboratory. Illumina HBV whole genome sequencing was performed in Oxford with a previously described protocol [9], with the addition of micrococcal nuclease for host nucleic acid depletion and sequencing on Illumina Miseq v3 with 2x300bp paired end reads. Bioinformatic workflow as described previously [9].

Supplementary Table 1: HBV drug resistance report from a clinical diagnostic virology laboratory, based on a serum sample submitted during rebound (year 25).

Polymorphisms in HBV reverse transcriptase (RT) codons 91-285 are listed, and summary of the interpretation of the resistance profile as reported by clinical diagnostic virology laboratory (based on https://hbv.geno2pheno.org/).

Genotype	Polymorphisms in RT domain	Clinical interpretation of resistance associated mutations
Dual infection with genotypes A and G	I53IS, T54A, I103V, N118D, I121S, N122H/I/L, N123D, N124H, M129L, W153R, V163I, A181T, V207I/M, L217R, I253V, V266A/I/T	• Lamivudine – resistant (181T)<br>• Telbivudine - resistant (181T)<br>• Adefovir – resistant (181T)<br>• Entecavir – susceptible<br>• Tenofovir - susceptible

Supplementary Table 2: Resistance associated mutations (RAMs) listed in EASL guidance [7], Genotype A/G consensus and polymorphisms identified in HBV isolated from this individual.

RAM	**Reference sequence** (NB. same aa for Geno A/G at all given sites)	**Patient HBV sequence** (UCLH report)	**Patient HBV sequence (Illumina data)**		**Clinical interpretation from EASL guidance (if detected)**			
			**Consensus**	**Minority variant**	**LAM**	**ETV**	**ADV**	**TDF/ TAF***
I169T (in combination)	I		I (100%)					
V173L (in combination)	V		V (99%)	G (1%)				
L180M	L		L (100%)					
A181T/V	A	A, T	A (97%)	T (3%)	I	S	R	I
T184G (in combination)	T		T (100%)					
S202I/G (in combination)	S		S (100%)					
M204V/I	M		M (100%)					
N236T	N		N (100%)					
M250V (in combination)	M		M (100%)					

The amino acid substitution profiles are shown in the left column and the level of susceptibility is given for each drug: S (sensitive), I (intermediate/reduced susceptibility), R (resistant).

ETV, entecavir; TDF tenofovir disoproxil fumarate; TAF, tenofovir alafenamide; LAM, lamivudine; ADV, adefovir

*In vitro data for tenofovir, in vivo data for TDF, no clinical data for TAF.
